# Imaging of labile Fe^2+^ and Fe^3+^ in living *Arabidopsis thaliana* roots

**DOI:** 10.1093/plphys/kiae221

**Published:** 2024-04-22

**Authors:** Carine Alcon, Arnaud Comte, Catherine Curie, Tou Cheu Xiong

**Affiliations:** IPSiM, Univ Montpellier, CNRS, INRAE, Montpellier, France; Univ Lyon, Université Claude Bernard Lyon 1, Villeurbanne, France; IPSiM, Univ Montpellier, CNRS, INRAE, Montpellier, France; IPSiM, Univ Montpellier, CNRS, INRAE, Montpellier, France

## Abstract

Adapting fluorescent iron imaging to living plants enables visualizing labile Fe^2+^ and Fe^3+^ pools, revealing the heterogeneous distribution of iron redox status at the tissue and cellular levels.

Dear Editor,

Imaging of iron (Fe) in living organisms is challenging, and ways to visualize Fe are limited to sophisticated elemental methods and/or fixed tissues. As a transition metal, Fe cycles between 2 oxidation states, Fe^2+^ and Fe^3+^, losing or donating an electron in doing so. This property enables Fe to participate in key metabolic pathways (Briat et al. 2015). Imaging of the redox species of Fe is therefore of interest to decipher its biological functions. Cellular Fe is partitioned into 2 distinct pools (Koppenol and Hider 2019): static Fe, which is tightly bound to its ligands, and labile Fe, which is weakly bound and can be exchanged between ligands rather effortlessly. To date, there are no reports describing the distribution of Fe^2+^ and Fe^3+^ labile pools in living organisms. The Perls-DAB histochemical method stains Fe in fixed tissues, chiefly the Fe^3+^ form (Roschzttardtz et al. 2009), but it mainly detects the static Fe fraction since labile Fe is likely lost during tissue fixation. In order to address the dynamics of Fe^2+^ and Fe^3+^ labile pools in live plants, we have established a method combining 2 probes, which enables specific detection of the redox state of the labile Fe pools.

To that aim, we have selected 2 fluorescent probes, SiRhoNox-1 (Hirayama et al. 2017) and MPNBD (Park et al. 2014), which we used to image labile Fe^2+^ and Fe^3+^, respectively, in Arabidopsis (*Arabidopsis thaliana*) roots (Supplementary Methods). The 2 probes were chosen in such a way that their spectral properties do not overlap, allowing their simultaneous utilization without any crosstalk (Supplementary Fig. S1). The specificity of each probe was reconfirmed in vitro albeit in an aqueous buffer adapted for plant applications (Supplementary Fig. S2). The fluorescence of the 2 probes, though depending on pH, was found rather stable at physiological pH (6.0 to 6.5; Supplementary Fig. S2, G and H). Fluorescence intensity fluctuations must therefore be interpreted cautiously. The selectivity of the probes toward the redox state of Fe was tested by applying them to various Fe species in vitro. The mixed probes detected labile Fe species (Fe(II)-acetate, Fe(II)-SO_4_, Fe(III)-NO_3_, and Fe(III)-Cl_3_), but not the Fe species involved in strong chelates such as EDTA or citrate (Fig. 1A). In vitro ascorbate-mediated reduction of Fe^3+^ species into Fe^2+^ was successfully monitored by the probes (Fig. 1B), suggesting that the method is suitable to assess reductase activity in vivo.

**Figure 1.**
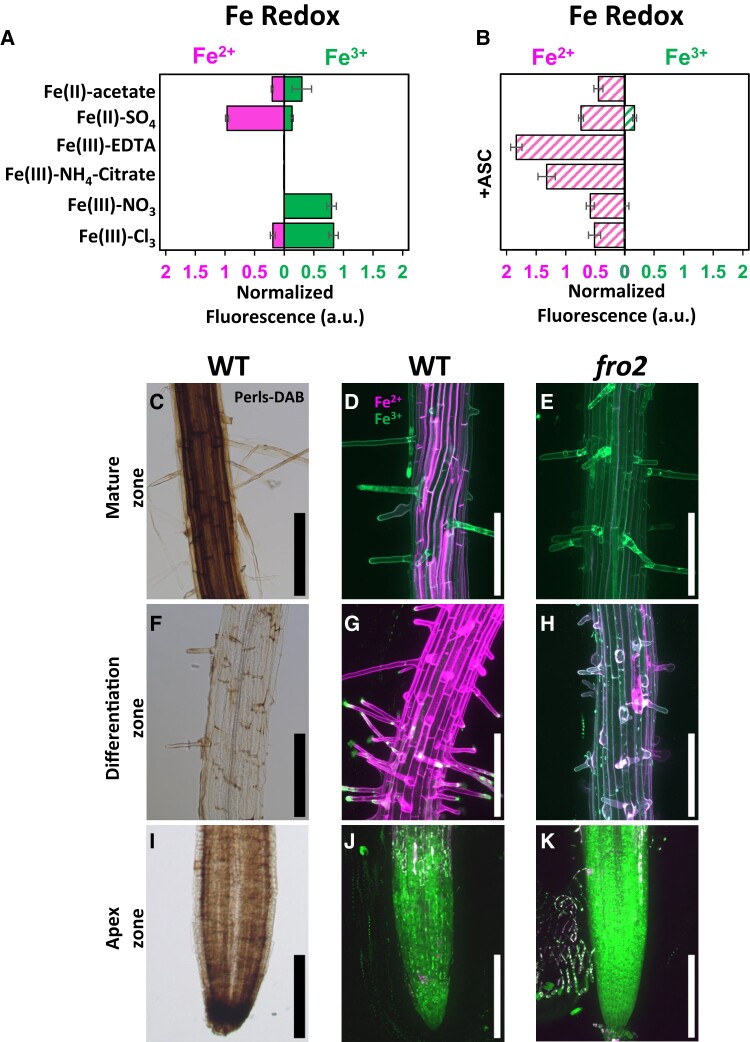
In vitro and in vivo detection of labile Fe^2+^ and Fe^3+^ using fluorescent probes. **A, B)** Determination of the specificity of the SiRhoNox-1 and MPNBD probes in vitro. **A)** Both probes were applied in combination to 1 mM solutions of a variety of Fe species. **B)** Reduction of Fe^3+^ by addition of 1 mM ascorbate (ASC) to the different Fe species allowed detecting a change of Fe redox state. **C to K)** In vivo imaging of Fe in the roots of 7-d-old Arabidopsis plants grown on 0.5xMS containing 50 *µ*M Fe-EDTA. Labile Fe, detected using combined SiRhoNox-1 and MPNBD **D**, **E, G, H, J, K)**, was compared with Fe histochemical staining with the Perls-DAB method **C, F, I)**. In the primary root, the mature zone **C to E)**, differentiation zone **F to H)**, and apex zone **I to K)** are shown. The projection of maximum intensity of the Z-stack of fluorescent pictures is shown for SiRhoNox-1 (Fe^2+^, magenta LUT) and MPNBD (Fe^3+^, green LUT) in wild-type (WT) plants **D, G, J)** and in the *fro2* mutant **E, H, K)**. Data shown are mean ± Sd. Data were collected from 3 to 4 independent experiments. a.u., arbitrary unit; DAB, 3,3′-diaminobenzidine. All scale bars = 200 *µ*m.

SiRhoNox-1 and MPNBD were applied in combination to 7-d-old plants and compared with Perls-DAB staining (Fig. 1, C to K). The fluorescent signals observed with the 2 probes were heterogeneously distributed along the entire primary root and were distinct from each other (Supplementary Fig. S3). Three root zones representative of the distribution of Fe^2+^ and Fe^3+^ were observed at higher magnification (Fig. 1, D, G, and J). Fe^3+^ was markedly predominant in the primary root apex (Fig. 1J) but absent in the young lateral root (Supplementary Fig. S4, A to C). Likewise, Perls-DAB did not stain the emerging root, suggesting that if Fe is present at this stage, its level is under the detection threshold of the 2 methods (Supplementary Fig. S4D). The primary root apex exhibited no Fe^2+^ signal (Fig. 1J). In contrast, in the differentiation zone, a strong Fe^2+^ fluorescent signal was observed at the cell periphery (Fig. 1G), suggesting an apoplastic localization. This observation is in agreement with previous studies reporting elemental analyses of cellular fractions (Bienfait et al. 1985; Ye et al. 2015; Liu et al. 2023). Plasmolysis of root cells confirmed the apoplastic localization of Fe^2+^ (Supplementary Fig. S5). Moreover, colocalization of FM4-64 and SiRhoNox-1 revealed the presence of Fe^2+^ at the plasma membrane (Supplementary Fig. S5F). 3D images of each root zone emphasized the variation of distribution of the 2 Fe redox species between cell layers and according to root age (Supplementary Fig. S6).

The Fe redox imaging method was applied to the Fe homeostasis ferric reduction oxidase 2 mutant (*fro2*), the Fe^3+^/Fe^2+^ ratio of which is imbalanced owing to a loss of its ability to reduce Fe^3+^ at the root surface (Robinson et al. 1999; Connolly et al. 2003). Compared to wild type, the differentiation and mature zones of the *fro2* root expectedly exhibited a dramatic decrease of fluorescence with SiRhoNox-1, confirming the specificity of the SiRhoNox-1 probe for Fe^2+^ in vivo (Fig. 1, E and H, Supplementary Fig. S7). The penetration ability of the probes was examined using confocal microscopy. Fluorescence of SiRhoNox-1 and MPNBD was visible in most layers of the root including the vascular cylinder (Fig. 2, A to E), indicating that the 2 probes are able to penetrate all the tissues of the root. In addition, fluorescence signals of the Fe probes were detected inside the cells, showing the permeability of the plasma membrane toward these probes. In epidermal cells, MPNBD fluorescence filled the symplast (Fig. 2, F and H), whereas SiRhoNox-1 fluorescence surrounded the cells (Fig. 2G). Upon Fe limitation, SiRhoNox-1 produced intracellular punctuate signals (Fig. 2, I and J; Hirayama et al. 2013, 2017). Interestingly, the method highlighted a polarized pattern of Fe^2+^ at the external side of epidermal cells in the differentiation zone (Fig. 2, K to M), a feature that had not been reported previously. Such Fe^2+^ polarization is reminiscent of the polar localization of FRO2 in the same cell type (Martín-Barranco et al. 2020). Remarkably, this polar distribution shifted to the inner side of the epidermal cells in the mature zone (Fig. 2, compare K-O and P-T) where Fe^2+^ was observed in the apoplast (Fig. 2T, red arrows). Quantification of the fluorescence signals allowed detecting subtle changes in the balance between Fe states, as shown in Fe-sufficient and Fe-deficient conditions in wild type and the *fro2* mutant (Supplementary Fig. S7).

**Figure 2.**
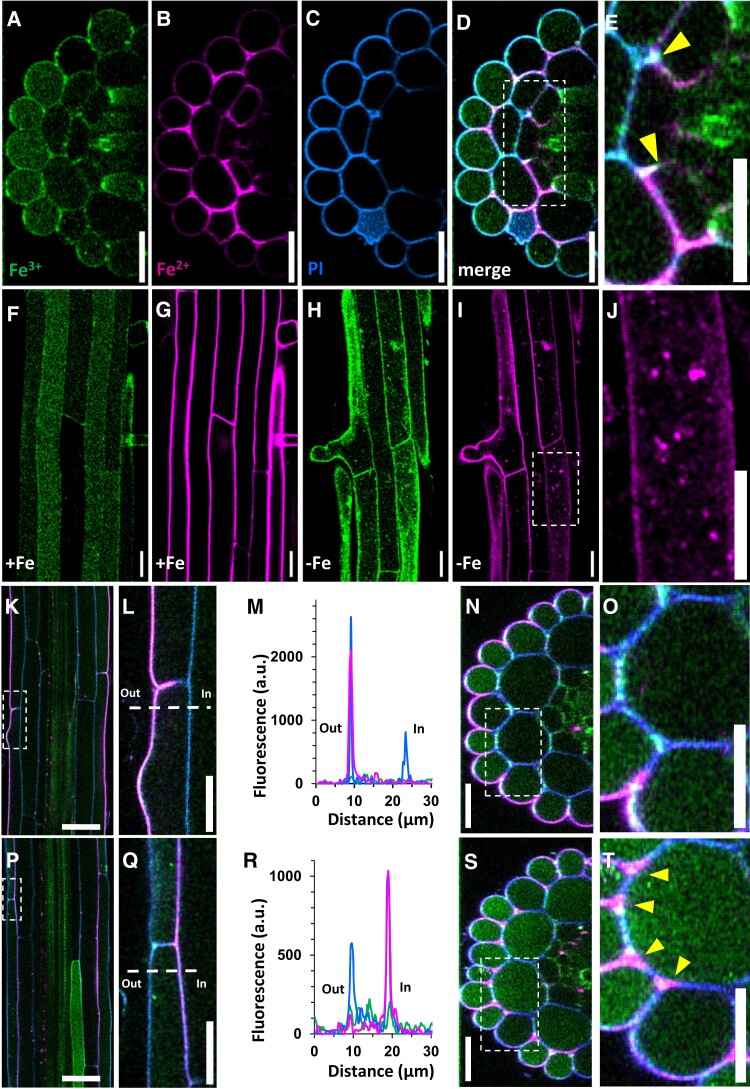
Distribution of labile Fe in the different cell layers of the primary root of *A. thaliana*. Images were taken from 7-d-old seedlings. **A to E)** Representative orthogonal view of the mature zone of the primary root stained with MPNBD (Fe^3+^, **A**), SiRhoNox-1 (Fe^2+^, **B**), propidium iodide (cell wall, **C)**, or a merged image of the 3 probes **D, E)**. Labile Fe^2+^ and Fe^3+^ are present in most cell types with a signal in the endodermis. An enlarged view of the endodermal layer indicated by a dashed square shows interruption of the apoplastic fluorescent signal at the Casparian strip (arrowheads, **E**). **F to J)** MPNBD and SiRhoNox-1 fluorescent signals in root epidermis showing the presence of Fe^3+^ inside the cell **F)** and Fe^2+^ in the apoplast **G)** of Fe-replete plants as well as in intracellular dot-like structures of Fe-deficient plants **H to J)**. **J)** Close-up view of the Fe^2+^ dots presented in **I)**. **K to T)** Polar distribution of Fe^2+^ in the epidermal cell wall. Differentiation **K to O)** and mature **P to T)** zones of the primary root were observed in longitudinal sections. Higher magnification of the epidermal cells **L, Q)** shows polar localization of Fe^2+^, albeit in opposite pattern in the 2 zones, which is confirmed by the line profile of the fluorescence intensity of the probes **M, R)**. **N**, **S**, **O, T)** Orthogonal view of the differentiation **N, O)** and mature **S, T)** root zones, including the corresponding enlarged views **O, T)** indicated by dashed areas within **N) and S)**. **T)** SiRhoNox-1 labels the intercellular space in the mature zone (red arrows). **A to G** and **K to T)** Seedlings were grown on 0.5xMS containing 50 *µ*M Fe-EDTA (+Fe). **H to J)** Seedlings were grown on 0.5xMS without Fe (−Fe). Magenta LUT: SiRhoNox-1; green LUT: MPNBD; BIOP-Azure LUT: propidium iodide. MPNBD, 7-(4-methylpiperazin-1-yl)-4-nitrobenzo-2-oxa-1,3-diazole; a.u., arbitrary unit. All scale bars = 20 *µ*m.

In summary, combining fluorescent probes for Fe^2+^ and Fe^3+^ represents an original method to distinguish the redox species of Fe within live tissues, reveals their distribution in root, and uncovers a remarkable polarization of Fe^2+^. Because this method can detect subtle differences of Fe charges in the tissues, it will become useful to characterize actors of the redox status of Fe, such as oxidoreductases, hence equipping the community with a powerful tool to explore Fe homeostasis in plants.

## Supplementary Material

kiae221_Supplementary_Data

## Data Availability

The data underlying this article will be shared on reasonable request to the corresponding author.

## Supplementary data

The following materials are available in the online version of this article.

**Supplementary Figure S1.** Excitation and emission spectra of SiRhoNox-1 and MPNBD probes at pH 6.0.

**Supplementary Figure S2.** In vitro characterization of the Fe redox SiRhoNox-1 and MPNBD fluorescent probes.

**Supplementary Figure S3.** Distribution of Fe along the primary root of *A. thaliana* grown on 0.5xMS containing 50 *µ*M Fe-EDTA.

**Supplementary Figure S4.** The lateral root apex is not stained by the two Fe probes.

**Supplementary Figure S5.** Fe is localized in the apoplastic space of root epidermal cells.

**Supplementary Figure S6.** Differential spatial distribution of Fe^2+^ and Fe^3+^ according to the developmental stage of the root in 7-d-old plants grown in Fe-replete conditions.

**Supplementary Figure S7.** Changes in Fe redox state are dependent on growth conditions and Fe homeostasis.

## Dive Curated Terms

The following phenotypic, genotypic, and functional terms are of significance to the work described in this paper:

FRO2 Gramene: AT1G01580
FRO2 Araport: AT1G01580
