# Supplementary material for: Imaging of labile Fe2+ and Fe3+ in living Arabidopsis thaliana roots
Source: Plant Physiol. 2024 Apr 22;195(4):2520–3. doi: 10.1093/plphys/kiae221 (PMC11288730; doi:10.1093/plphys/kiae221)
Supplement: kiae221_Supplementary_Data [file kiae221_supplementary_data.pdf]

Supplementary Figure S1

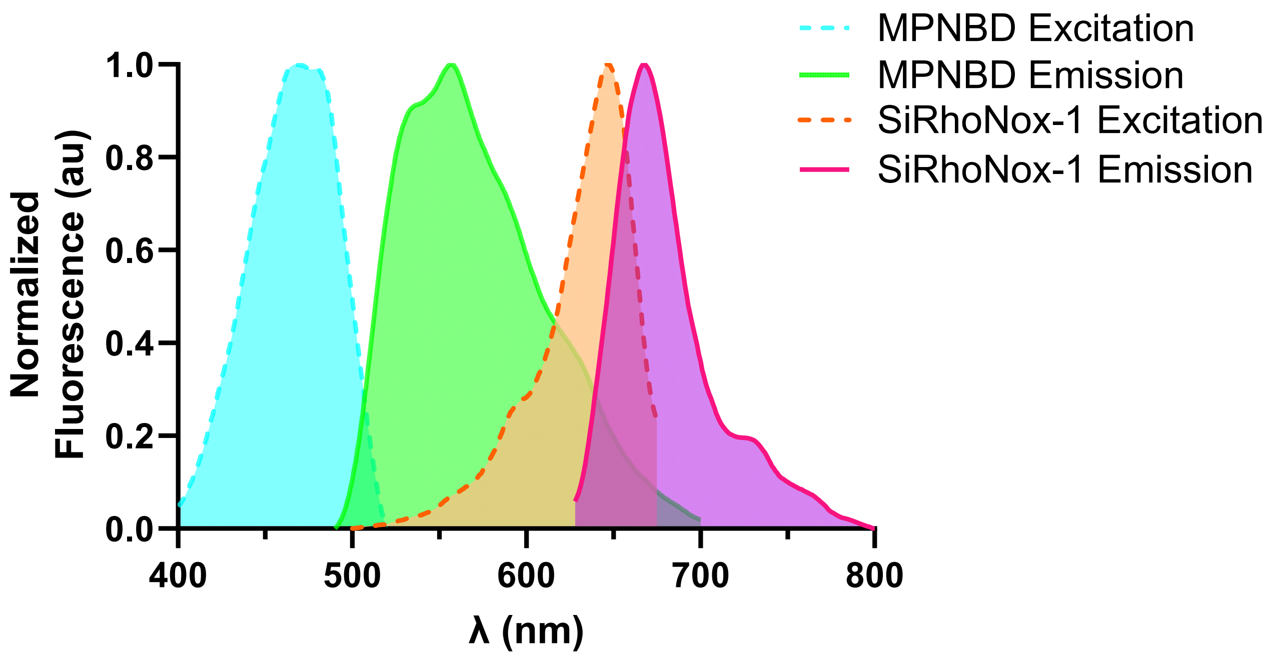

Supplemental Figure S1: Excitation and emission spectra of SiRhoNox-1 and MPNBD probes at pH 6.0. Fluorescence excitation and emission spectra of 1 µM MPNBD and 0.5 µM SiRhoNox-1 in the presence of 1 mM $FeCl_3$ or 50 µM $FeSO_4$ respectively, were determined with the spectrofluorimeter Clariostar plus (BMG). The spectra were normalized to show the maximum excitation and emission for both probes at pH 6.0. MPNBD = 7-(4-methylpiperazin-1-yl)-4-nitrobenzo-2-oxa-1,3-diazole; a.u.= arbitrary unit.

Supplementary Figure S2

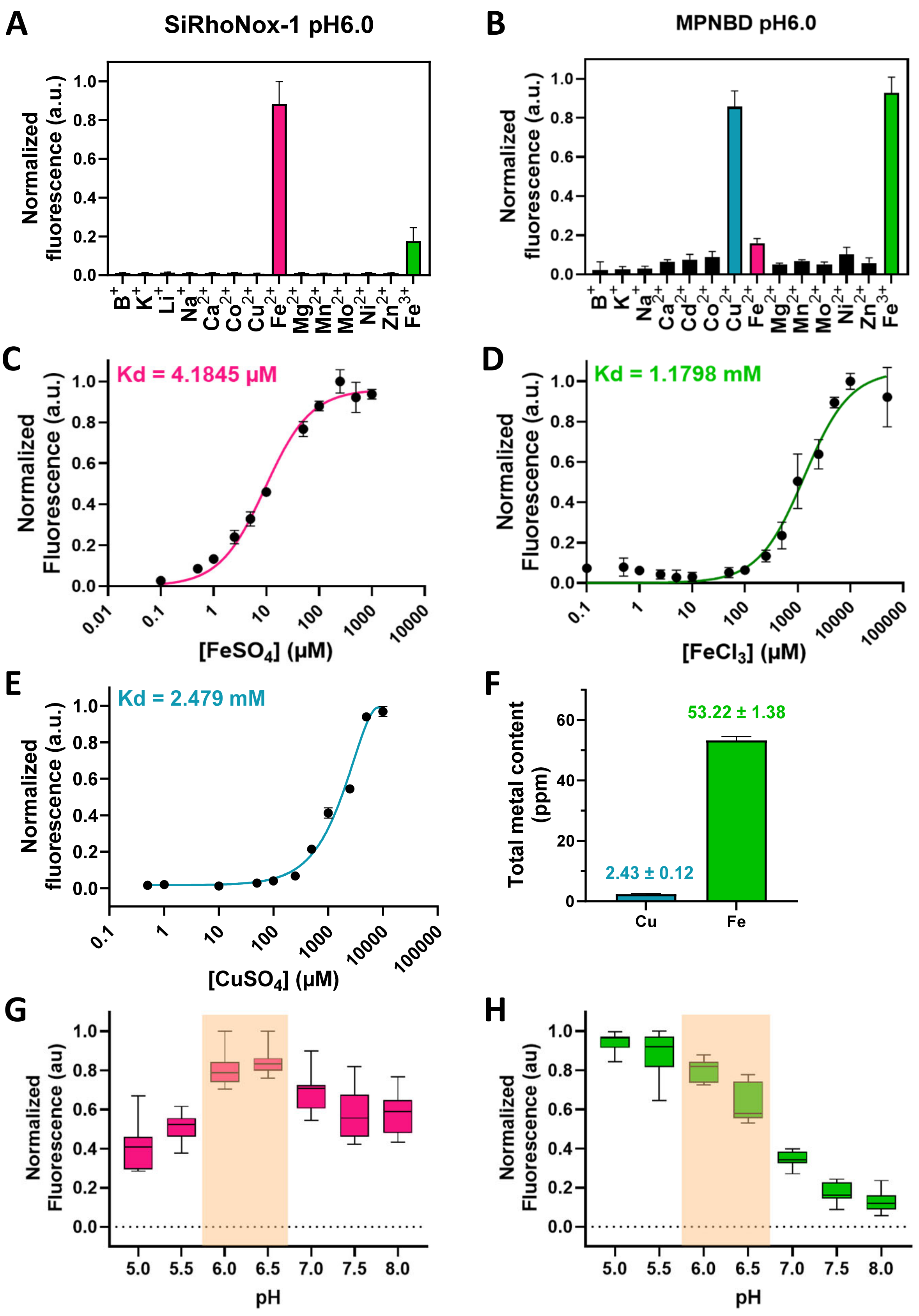

Supplemental Figure S2: *In vitro* characterization of the Fe redox SiRhoNox-1 and MPNBD fluorescent probes. (**A**, **B**)**:** Selectivity of the probes for Fe^{2+} and Fe^{3+}. Cations were incubated with the probes at pH 6.0 and fluorescence was measured for SiRhoNox-1 (**A**) and MPNBD (**B**). (**C**, **D**): Titration curves for $FeSO_4$ (**C**), $FeCl_3$ (**D**) and $CuSO_4$ (**E**) allowed calculating the apparent affinities (Kd) for each fluorescent probe at pH 6.0. (**F**) Total Cu and Fe measured by ICP-OES from Arabidopsis roots. (**G**,**H**): pH-dependent fluorescence of the probes calculated using 100 µM $FeSO_4$ for SiRhoNox-1 (**G**) and 1 mM $FeCl_3$ for MPNBD (**H**). The physiological pH range [6.0-6.5] is depicted in orange (**G**,**H**). All data shown are mean ± SD of the normalized fluorescent values. Data were collected from 3 independent experiments of 3-6 replicates each. Data for Fe^{3+}, Fe^{2+} and Cu^{2+} are shown in green, magenta and cyan respectively. Whickers of box plots show the min and max values. MPNBD = 7-(4-methylpiperazin-1-yl)-4-nitrobenzo-2-oxa-1,3-diazole; a.u.= arbitrary unit; Kd = dissociation constant.

Supplementary Figure S3

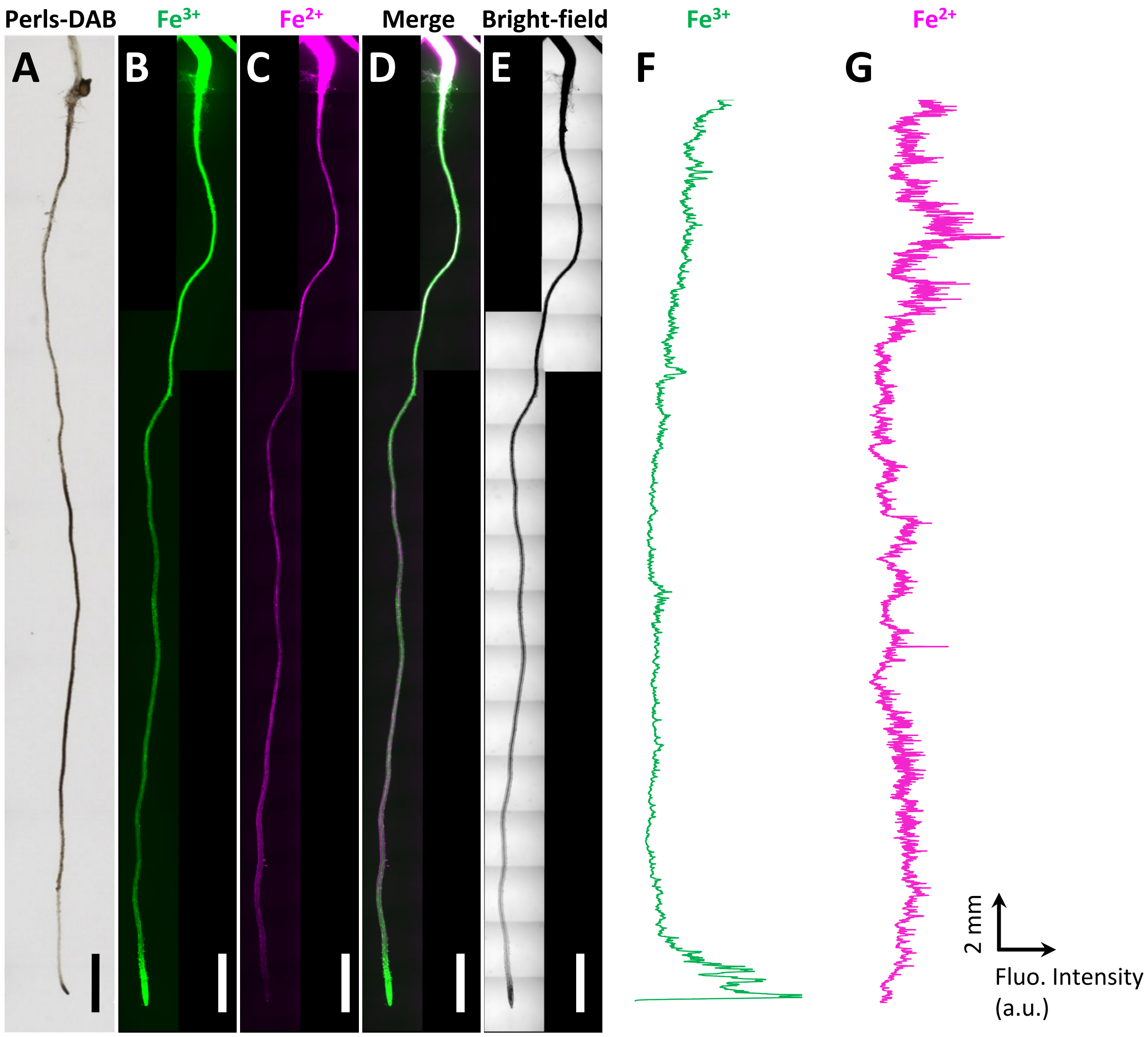

Supplemental Figure S3: Distribution of Fe along the primary root of A. thaliana grown on 0.5xMS containing 50 µM Fe-EDTA. The primary root of 7 day-old *in vitro* grown plants was stained with Perls-DAB (**A**), or a combination of MPNBD and SiRhoNox-1 (**A**-**D**). The fluorescence mosaic image is shown for either MPNBD (**B**), or SiRhoNox-1 (**C**), or both MPNBD and SiRhoNox-1 (**D**). The bright-field mosaic image is shown in (**E**). The two methods show heterogeneous distribution of Fe in the primary root. Merge of the fluorescence images show different distributions of Fe^{2+} and Fe^{3+} along the primary root (**D**). (**F**, **G**): Relative quantification of Fe^{3+} (**F**, green curve) and Fe^{2+} (**G**, magenta curve) along the root. MPNBD = 7-(4-methylpiperazin-1-yl)-4-nitrobenzo-2-oxa-1,3-diazole; a.u. = arbitrary unit; DAB= 3,3'-Diaminobenzidine. All scale bars = 2 mm.

Supplementary Figure S4

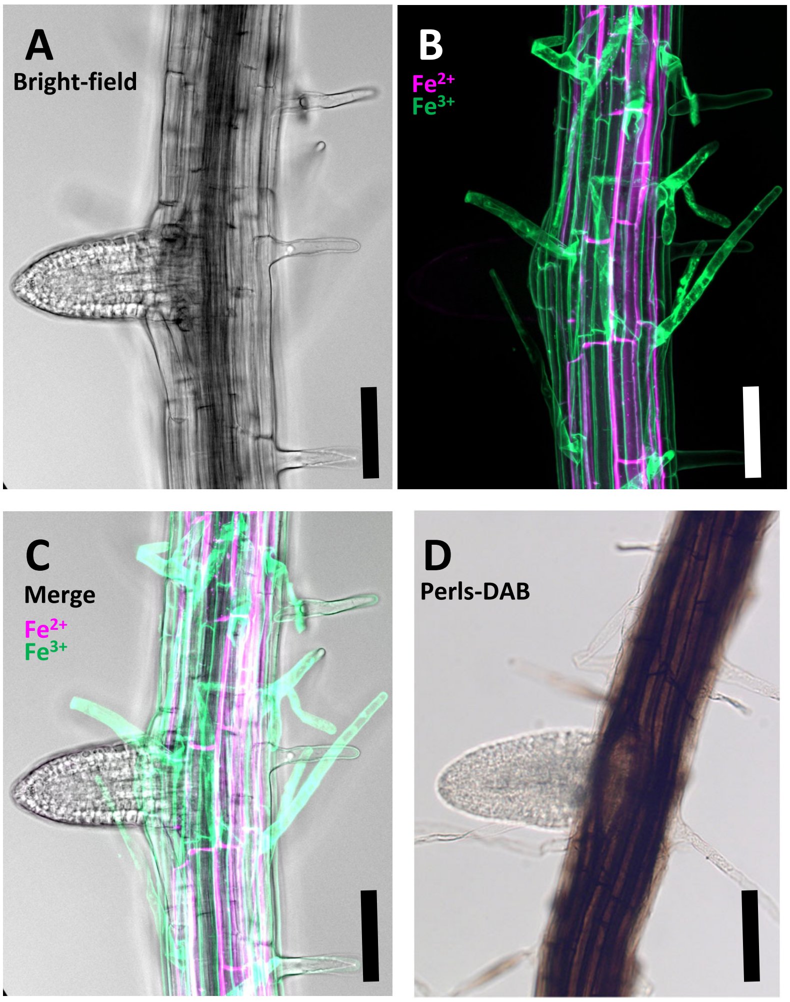

Supplemental Figure S4: The lateral root apex is not stained by the two Fe probes. (**A**): Bright-field image of the root showing emergence of a lateral root. (**B**): Maximal projection of Z-stack images of a 7 day old root in the mature zone stained with a combination of MPNBD (Fe^{3+}) and SiRhoNox-1 (Fe^{2+}). (**C**): Merge of the bright-field and fluorescence images. (**D**): Perls-DAB staining of a lateral root. MPNBD = 7-(4-methylpiperazin-1-yl)-4-nitrobenzo-2-oxa-1,3-diazole; DAB= 3,3'-Diaminobenzidine. All scale bars = 100 µm.

Supplementary Figure S5

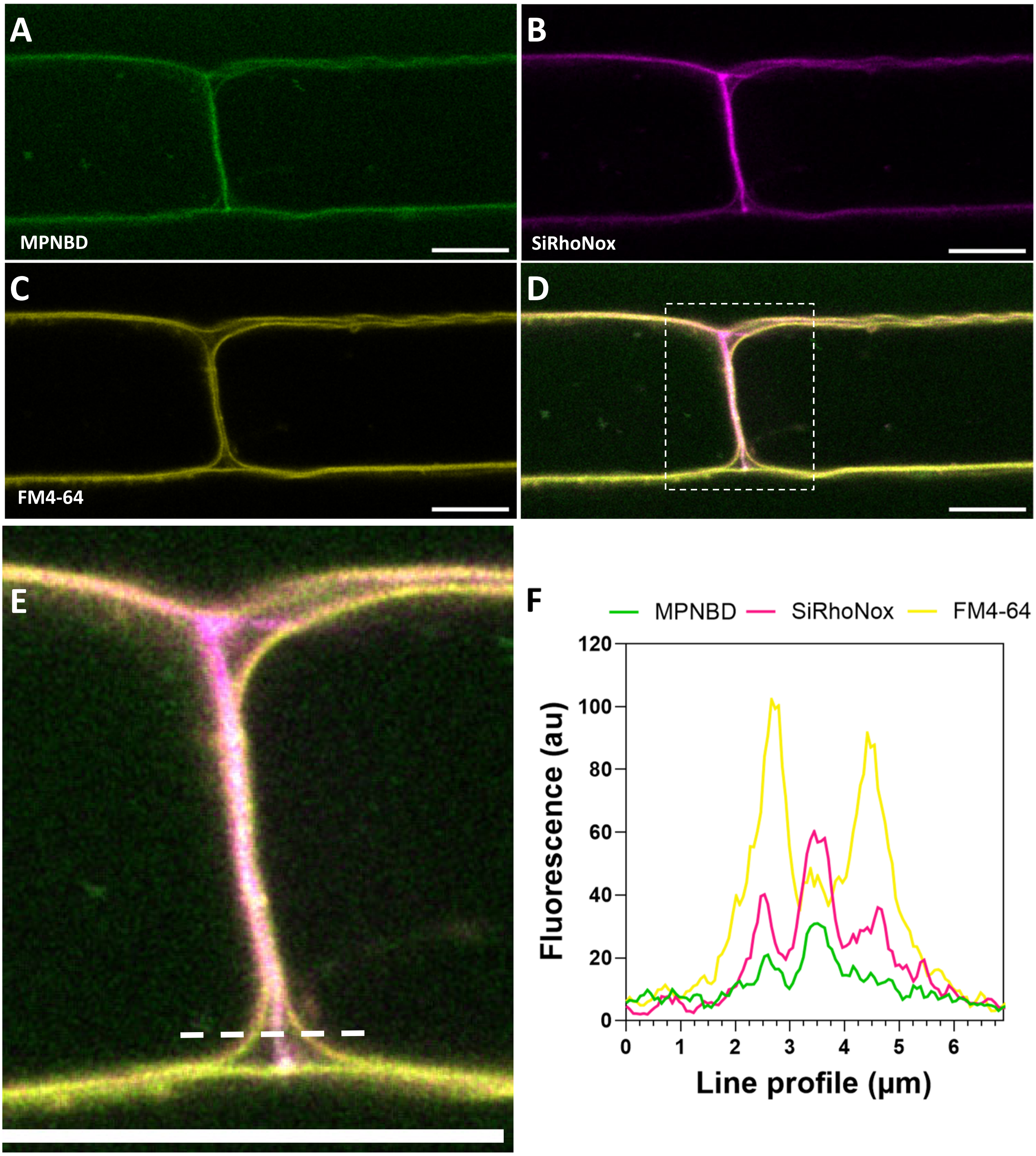

Supplemental Figure S5: Fe is localized in the apoplastic space of root epidermal cells. (**A**-**D**): 7 day-old Arabidopsis plants grown on 0.5xMS containing 50 µM Fe-EDTA were stained with a combination of 10 µM MPNBD (**A**, Fe^{3+}, green), 1 µM SiRhoNox-1 (**B**, Fe^{2+}, magenta) and 4 µM FM4-64 (**C**, Plasma membrane, yellow) prior to being plasmolyzed using 300 mM sorbitol. (**D**) Merged image of the three fluorescent signals shown in (**A**), (**B**) and (**C**). (**E**) Close-up view of the merged image corresponding to the area indicated by a dashed square in (**D**). (**F**) Intensity profile of the three probes across the junction between two neighbor cells, along a dash line indicated in (**E**). These observations indicate that Fe^{2+} localizes at the apoplast, and to a lesser extent at the plasma membrane as SiRhoNox-1 also co-localizes with FM4-64 (**D**, **E**, **F**). MPNBD = 7-(4-methylpiperazin-1-yl)-4-nitrobenzo-2-oxa-1,3-diazole; a.u.= arbitrary unit; All scale bars = 10 µm.

Supplementary Figure S6

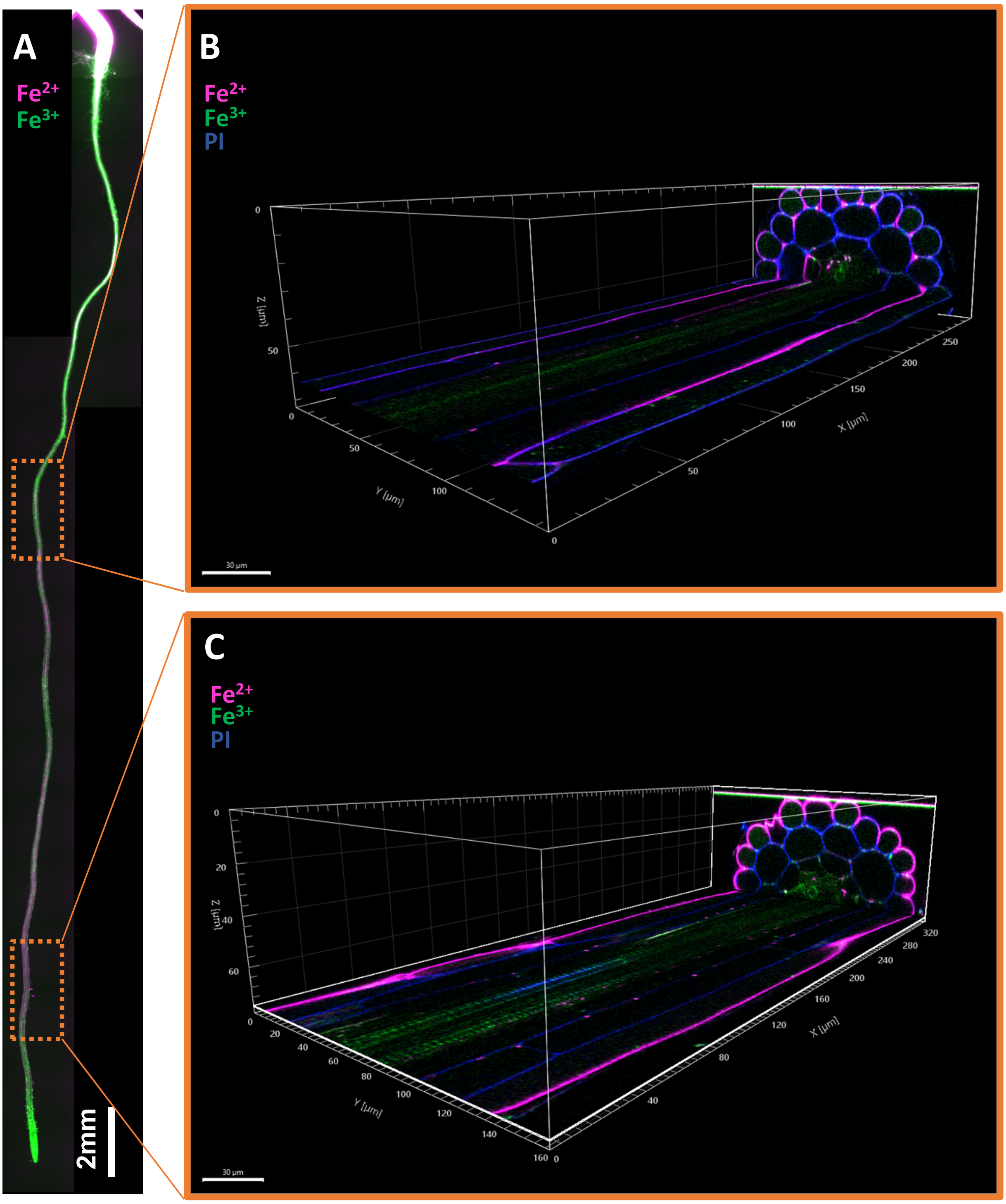

Supplemental Figure S6: Differential spatial distribution of Fe^{2+} and Fe^{3+} according to the developmental stage of the root in 7 day-old plants grown in Fe replete conditions. (**A**): Position of the two zones of the primary root was indicated on the mosaic image shown in Fig. S3D. (**B-C**): 3D views of Fe^{2+} and Fe^{3+} distribution in the mature zone (**B**), the differentiation zone (**C**) of primary root stained with SiRhoNox-1 (Fe^{2+}: magenta), MPNBD (Fe^{3+}: green) and propidium iodide (Cell wall, blue).

Supplementary Figure S7

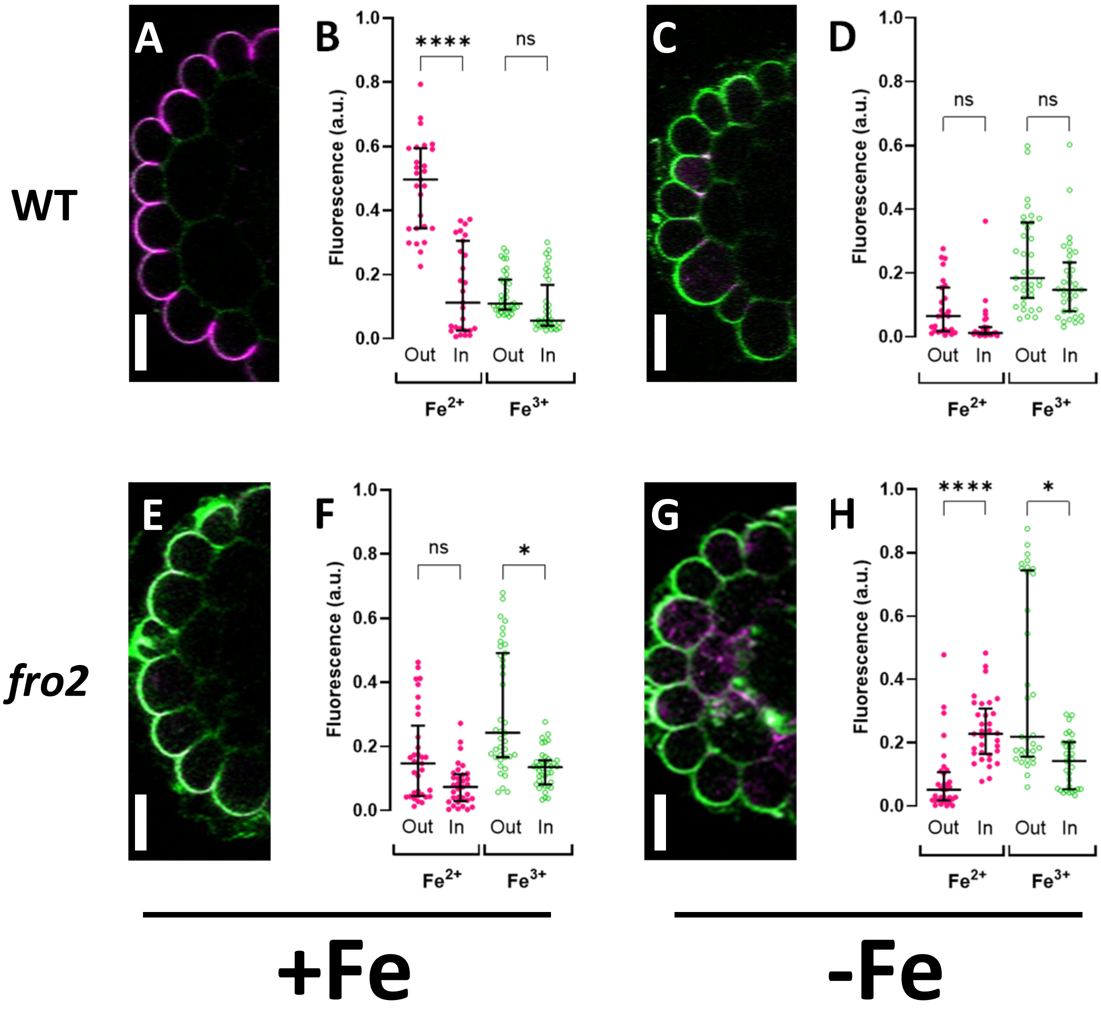

Supplemental Figure S7: Changes in Fe redox state are dependent on growth conditions and Fe homeostasis. Orthogonal merged views of the differentiation zone of the primary root of 7 day-old wild-type (WT) Arabidopsis (**A**, C) or *fro2* mutant (**E**, **G**) plants grown in the presence (**A**, **E**) or absence (**C**, **G**) of Fe (50µM Fe-EDTA) and stained with both SiRhoNox-1 and MPNBD. Quantification of the signal of the two fluorescent probes is shown in each condition for the outer (Out) and inner (In) side of the epidermal cells (**B**, **D**, **F**, **H**). Fe deprivation decreased the amount of Fe^{2+} at the outer side of epidermal cells for both WT (**C**, **D**) and fro2 roots (**G**, **H**). The lack of Fe^{3+} reductase activity in the *fro2* mutant decreased labile Fe^{2+} and increased labile Fe^{3+}. All scale bars = 20 µm. Data are representative of 3-4 independent experiments. All significant results were calculated following the non parametric Kruskal-Wallis test. P values <0.0001 and <0.01 are indicated with **** and *, respectively. ns = non significant. Horizontal bars represent median and interquartile range values. a.u.= arbitrary unit.

Supplementary Methods

Chemicals Fe probes

MPNBD (7-(4-methylpiperazin-1-yl)-4-nitrobenzo-2-oxa-1,3-diazole) was synthesized according to Park et al. (2014). 4-chloro-7-nitrobenzofurazan (3.2 g, 16 mmol) was dissolved in $CHCl_3$ (100 mL) and 1-methylpiperazine (1.87 mL, 16.8 mmol) was added. The reaction was stirred for 30 min at room temperature and TLC analysis showed residual starting material. More amine was added (0.2 ml) and the mixture was stirred for 20 min to obtain total conversion. The reaction mixture was concentrated on a rotary evaporator and the residue was purified by column chromatography (SiO_2, CH_2Cl_2:MeOH 95:5) to afford 3.9 g of product. This product was dissolved in minimum $CHCl_3$ and Et_2O was added. The resulting slurry was cooled to 0°C, filtered and washed with Et_2O to obtain MPNBD as an orange solid (3.1g, 74%). ^{1}H-NMR (300 MHz, $CDCl_3$): δ 2.42 (s, 3H), 2.70 (m, 4H), 4.15 (m, 4H), 6.30 (d, 1H, J = 8.8 Hz), 8.41 (d, 1H, J = 8.8 Hz). HRMS (ESI) calculated for $[M+H]^+$ $C_{11}H_{14}N_5O_3$: 264.1091, observed for 264.1090.

The MPNBD was dissolved in DMSO at 10 mM and stored at -20°C. SiRhoNox-1 (Hirayama et al., 2017, commercial name: FerroFaRed, Goryo Chemicals) was dissolved in DMSO at 1 mM and was stored at -20°C

Growth conditions and plant materials

Wild type columbia-0 Arabidopsis (*Arabidopsis thaliana*) and the *fro2* mutant (GK-055E10) were grown vertically on half-strength Murashige and Skoog (0.5xMS) with agar (0.7% w/v) medium supplemented with 1 % (w/v) sucrose and 2.5 mM MES-KOH to pH 5.7 and containing Fe (+Fe: 50 µM Fe-EDTA) or not (-Fe) as indicated in the legend of the figures, for 7 days at 21°C in a 16-h light/8-h dark cycle with 65% relative humidity and a light intensity of 150 µmol.m^{-2}.s^{-1}

Microscopy

For wide-field microscopy, the observations were performed in 3 areas along the root of 7 day-old seedlings: apex zone, differentiation zone and mature zone, using a plan-apochromatic 20 X / 0.8 objective (Observer 7 microscope Zeiss with Light source X-Cite 120LEDmini (Excelitas)) and the following sets of filters: Ex BP 470/40, BS 495, Em 525/50 for MPNBD and Ex BP 640/30, BS 660, Em 690/50 for SiRhoNox-1. Z-stack pictures were acquired using the Zeiss Apotome module and allowed performing Z-max projections.

Mosaic pictures were taken to observe the entire primary root. For each tile, 10 slices of Z-stack every 1 µm were captured and the maximum projections were processed prior to stitching the tiles.

The images were captured with an OrcaFlash (Hamamastu) controlled with the Zen blue software (Zeiss).

For confocal microscopy, the observations were done with a confocal SP8 microscope (Leica) using a plan-apochromat 40x / 1.1 water objective. Z-stack acquisitions with the best axial resolution were taken with a sequential line mode according to the following setting for MPNBD, propidium iodide and SiRhoNox-1, respectively: Ex 488 nm (laser 12%)/ Em 500-550, Ex 561 nm (laser 3%)/ Em 580-630 nm, Ex 633 nm (laser 12%) / Em 650-700 nm. The high magnification images were taken at the best lateral resolution required for the line profile analysis. Prior to the observations, the seedlings were incubated for 3 hours with MPNBD and SiRhoNox-1 and then were transferred for 10 minutes in 2 µg.mL^{-1} propidium iodide.

***In vitro* characterization**

MPNBD (1µM) and SiRhoNox (0.5µM) probes were diluted in 10 mM MES-KOH pH 6.0 in the presence of various cations at 1 mM for MPNBD and 100 µM for SiRhoNox-1.

Different Fe chelates were used at 1 mM. Fe reduction was performed by adding 1 mM Ascorbate. The fluorescence intensity was monitored with a spectrofluorometer (Clariostar plus, BMG Labtech) according to the following protocol: excitation at 475/15 nm and 633/8 nm and emission at 550/15 nm and 660/8 nm for MPNBD and SiRhoNox-1, respectively. The intensity fold changes were expressed as normalized values. Data correspond to triplicates from three independent experiments.

Plant Fe staining with chemicals probes

7 day-old seedlings were incubated in 10 mM MES-KOH pH 6.0 and either 10 µM MPNBD or 1 µM SiRhoNox-1 for 3h in the dark at room temperature prior to observations under wide-field or confocal microscope.

Perls-DAB histochemical staining

Histochemical Fe staining by the Perls method with DAB intensification was performed according to Roschzttardtz et al. (2009) on 7 day-old seedlings and observations were performed using a BX61 Olympus microscope with the objective 20 X / 0.7. Images were acquired using a ProgRes C5 camera (Jenoptik).

Images quantification and processing

Quantification and processing of the images were done with FIJI software (Schindelin et al., 2012). The orthogonal projections were used for signal quantification. Outer and inner sides of epidermal cells were selected with a segmented line tool and average intensity values were extracted. The values were normalized with max and min corresponding to 255 and 0 fluorescence gray values respectively. The visualization of the 3D images was done with Imaris 9 (Oxford Instrument). Charts and statistical tests were performed with GraphPad Prism 10.

References

Hirayama T, Tsuboi H, Niwa M, Miki A, Kadota S, Ikeshita Y, Okuda K, Nagasawa H (2017) A universal fluorogenic switch for Fe(II) ion based on N-oxide chemistry permits the visualization of intracellular redox equilibrium shift towards labile iron in hypoxic tumor cells. Chem Sci **8**: 4858–4866

Park M-J, Jung H-S, Kim Y-J, Kwon Y-J, Lee J-K, Park C-M (2014) High-sensitivity fluorescence imaging of iron in plant tissues. Chem Commun **50**: 8547–8549

Schindelin J, Arganda-Carreras I, Frise E, Kaynig V, Longair M, Pietzsch T, Preibisch S, Rueden C, Saalfeld S, Schmid B, et al (2012) Fiji: an open-source platform for biological-image analysis. Nat Methods **9**: 676–682
